# AstroLogics: a simulation-based framework for the analysis of boolean model ensembles

**DOI:** 10.1093/bioinformatics/btag555

**Published:** 2026-07-25

**Authors:** Saran Pankaew, Vincent Noël, Loïc Paulevé, Denis Thieffry, Emmanuel Barillot, Laurence Calzone

**Affiliations:** Institut Curie, Université PSL, Paris F-75005, France; Université PSL, Mines Paris Tech, INSERM U1331, Paris F-75005, France; Institut Curie, Université PSL, Paris F-75005, France; Université PSL, Mines Paris Tech, INSERM U1331, Paris F-75005, France; LaBRI, CNRS UMR 5800, INP, Université de Bordeaux, Talence, France; Institut Curie, Université PSL, Paris F-75005, France; Université PSL, Mines Paris Tech, INSERM U1331, Paris F-75005, France; Département de Biologie, École Normale Supérieure, PSL Université, Paris, France; Institut Curie, Université PSL, Paris F-75005, France; Université PSL, Mines Paris Tech, INSERM U1331, Paris F-75005, France; Institut Curie, Université PSL, Paris F-75005, France; Université PSL, Mines Paris Tech, INSERM U1331, Paris F-75005, France

## Abstract

**Motivation:**

Boolean networks (BNs) have emerged as versatile tools for modeling cellular regulatory mechanisms due to their ability to capture key biological features despite their simplicity. Multiple BN synthesis methods have emerged in recent decades, aiming to infer BNs with dynamics that correspond to experimental data. Often, these methods generate multiple BN candidates or “model ensembles”. While these ensembles are valuable for representing cell populations and their heterogeneity, they are typically treated as a single component without examining their constituent features.

**Results:**

We present AstroLogics, a novel framework designed to analyze and identify differences in both dynamical behavior and logical regulation within a BN model ensemble. The framework calculates dynamical distances between BNs through exploration of their state transition graphs (STGs), enabling clustering of similarly functioning models that may represent different cellular fates or signaling mechanisms. AstroLogics also identifies key logical properties that govern each cluster, highlighting the core regulatory structures that differentiate model behaviors. Our approach leverages MaBoSS, a stochastic simulation tool that implements the Boolean Kinetic Monte-Carlo algorithm to address time interpretation in BNs. This probabilistic estimation method allows efficient probing of BN dynamics through stochastic simulations, overcoming the computational limitations of exhaustive STG analysis. Our framework also provides powerful visualization and classification of the BN ensemble. Through multiple use cases, we demonstrate how AstroLogics facilitates comprehensive analyses of model diversity and discovery of key regulatory structures within a BN ensemble.

**Availability and implementation:**

The AstroLogics package, along with tutorials and datasets, are available at https://github.com/sysbio-curie/AstroLogics.

## 1 Introduction

An important aim of current cellular and molecular biology research is to decipher the molecular mechanisms by which cells integrate diverse signals to control essential functions such as differentiation, proliferation, and stress response. Gaining insight into these decision-making mechanisms is crucial to uncover the molecular basis of diseases and developing targeted therapeutic strategies. However, because these processes are governed by complex, highly integrated, and parallel networks of interactions, computational models are indispensable tools for their study. Complex systems of interactions among genes, transcription factors, and other regulatory molecules that determine when and how genes are expressed, and ultimately control cellular decisions.

Various modeling approaches have been developed for gene regulatory networks (GRNs), including linear models, Bayesian networks, neural networks, differential equations, and stochastic models. Each approach offers different advantages depending on the biological detail required, computational complexity, and available data. Among these, Boolean networks (BNs) have emerged as particularly versatile and intuitive tools for modeling cellular regulatory mechanisms (see seminal studies by Sugita (1963), Kauffman (1969), and Thomas (1973)).

A BN represents genes and proteins as binary variables (active or inactive) and implements their regulatory relationships in terms of logical rules. Despite their simplicity, BNs can capture key features of biological systems, including attractors and robustness to noise. Their discrete nature makes them especially useful in situations where quantitative data is scarce, and their interpretability facilitates biological insight. As a result, BNs have gained wide adoption in systems biology and have been successfully applied to model processes such as signal transduction and cell fate determination (Grieco *et al*. 2013, Flobak *et al*. 2015, Collombet *et al.* 2017, Hemedan *et al*. 2022).

A recent challenge in the field of BN is network inference or synthesis, which aims to reconstruct BN models from experimental data and existing biological knowledge. Various methods have been developed to infer BNs from time-series data, using diverse algorithmic strategies (Liang *et al*. 1998, Lähdesmäki *et al.* 2003, Ostrowski *et al*. 2016, Chevalier *et al*. 2019). These approaches typically rely on biological constraints to reduce the search space. However, such constraints apply to a limited number of conditions or contexts, which generally leads to a high number of candidate models, referred to as a “BN model ensemble.”

The concept of ensemble modeling has gained momentum with the advances in machine learning, particularly with the use of gradient-boosted trees and random forests. This approach aims to capture both model uncertainty and potential model variability across different biological systems (e.g. different cells within a biological sample). The use of model ensembles spans a wide range of applications. For example, random BN ensembles have been employed to show emerging properties of families of BNs sharing properties related to their architecture or logic rules (Kauffman 2004, Krawitz and Shmulevich 2007). In another instance, BN ensembles were used to assess the dynamical properties of qualitative differential equations (Schwieger and Siebert 2017). In previous work, we demonstrated the use of BN ensembles to represent cell populations and account for their potential heterogeneity (Chevalier *et al.* 2020). We defined ensembles of BNs sampled from the multitude of models compatible with network architecture and predefined dynamical properties. Then, these models were simulated asynchronously and the simulations were aggregated by averaging.

This approach presents a significant limitation in that a model ensemble is typically treated globally without a thorough examination of the constituent BNs’ features. These features include similarities in BN dynamics and logical functions throughout the ensemble. Analyzing such characteristics is essential for evaluating the diversity within model ensembles, dynamical bifurcations in cell fates, and identifying key logical regulations that serve as core regulatory structures.

In order to supersede the current tool limitations, we present AstroLogics, a framework designed to compute dynamical distances between BNs, cluster models, and identify key logic regulations that characterize each cluster. We describe the core concepts of the framework and illustrate its application through several use cases.

## 2 Materials and methods

### 2.1 Introduction to Boolean modeling

A Boolean network (BN) consists of a *regulatory graph* (V,E) and *dynamical parameters* (K). The *regulatory graph* is a signed directed graph where $V=\{v_{1},v_{2},\ldots ,v_{n}\}$ represents *n* nodes (genes, proteins, complexes, etc.) and *E* represents signed directed edges showing interactions between components.

The *dynamical parameters* are represented by associating each $v_{i}$ with a discrete variable $D_{i}=\{0,1\}$, determined by logical functions $K={\left\{K_{i}\right\}}_{i=1,\ldots ,n}$. These functions use logical operators ∨ (OR), ∧ (AND), and ¬ (NOT) to define state transitions based on the regulation of the component states.

Time in Boolean modeling reflects the sequential nature of the state transitions. The most common update modes are: (i) the fully synchronous updating mode, where all components in the network are updated simultaneously, which amounts to a deterministic dynamics where each configuration has exactly one successor (Kauffman 1969); and (ii) the fully asynchronous updating mode, where only one node is updated in each transition, considering all possible elementary transitions, which amounts to a non-deterministic dynamics where a state can have multiple successors (Thomas 1973).

In this work, we focus on the fully asynchronous updating mode. As the information on the state changes (updating timing) is often unknown, this fully asynchronous updating mode offers a more flexible approach to capture overall dynamics.

### 2.2 Dynamical properties of Boolean models

Understanding the dynamics of the model requires an understanding of two key concepts: *states* and *transitions*. A state of the system is represented by a vector $s=(s_{1},s_{2},s_{3},\ldots ,s_{n})$ where *n* denotes the number of components, and $s_{i}$ the state of component $v_{i}$. The graph representing the whole solution space, including states and the possible transitions governed by the logic rules, is referred to as the state transition graph (STG), where nodes correspond to states and edges to transitions.

From the STG, multiple features can be extracted, including reachability and attractors. The terminal states of the STG are referred to as attractors, defined as terminal state sets with no escape transitions. Attractors come in two kinds: single state attractors (stable states), where component values remain constant, and cyclic attractors, which consist of sets of mutually reachable states with no outward transitions. These attractors have important biological significance. Single state attractors can represent cell differentiation states (Collombet *et al.* 2017) or final cellular fates (proliferation, death, senescent states, etc.) (Calzone *et al*. 2010), while cyclic attractors typically represent periodic behaviors such as cell cycles or circadian rhythms (Traynard *et al*. 2016).

When analyzing a model, we characterize its main dynamic properties, including attractors and their accessibility. While small models allow straightforward attractor identification through STG exploration, larger models with complex interactions face combinatorial explosion. This requires alternative methods to capture crucial dynamical information, such as attractors, reachability, basins of attraction, or trap spaces (Klarner *et al*. 2015).

### 2.3 Stochastic simulations of Boolean models

Computing STG transitions requires considerable computational power and the resulting graphs can be hard to display and interpret. To address this, a simulation-based approach enables an exploration of the STG through stochastic simulations. In this work, we use MaBoSS, a stochastic simulation tool for continuous-time Markov processes in C++ (Stoll *et al*. 2012, 2017). It relies on the Boolean Kinetic Monte-Carlo (BKMC) algorithm, assigning transition rates to each node activation and deactivation, thereby enabling the implementation of different timescales and the introduction of continuous time in the Boolean framework. The tool performs multiple random walks on the probabilistic STG, calculating the activation likelihood for each node and state. Users can modify parameters, such as initial conditions and node activity status, for a thorough analysis. The simulation produces a vector of activation probabilities for states in the STG, or for each node of the model over time. Further details on states and nodes activation probability are provided in the Supplementary Information, available as supplementary data at *Bioinformatics* online.

### 2.4 AstroLogics framework

**Figure 1:**
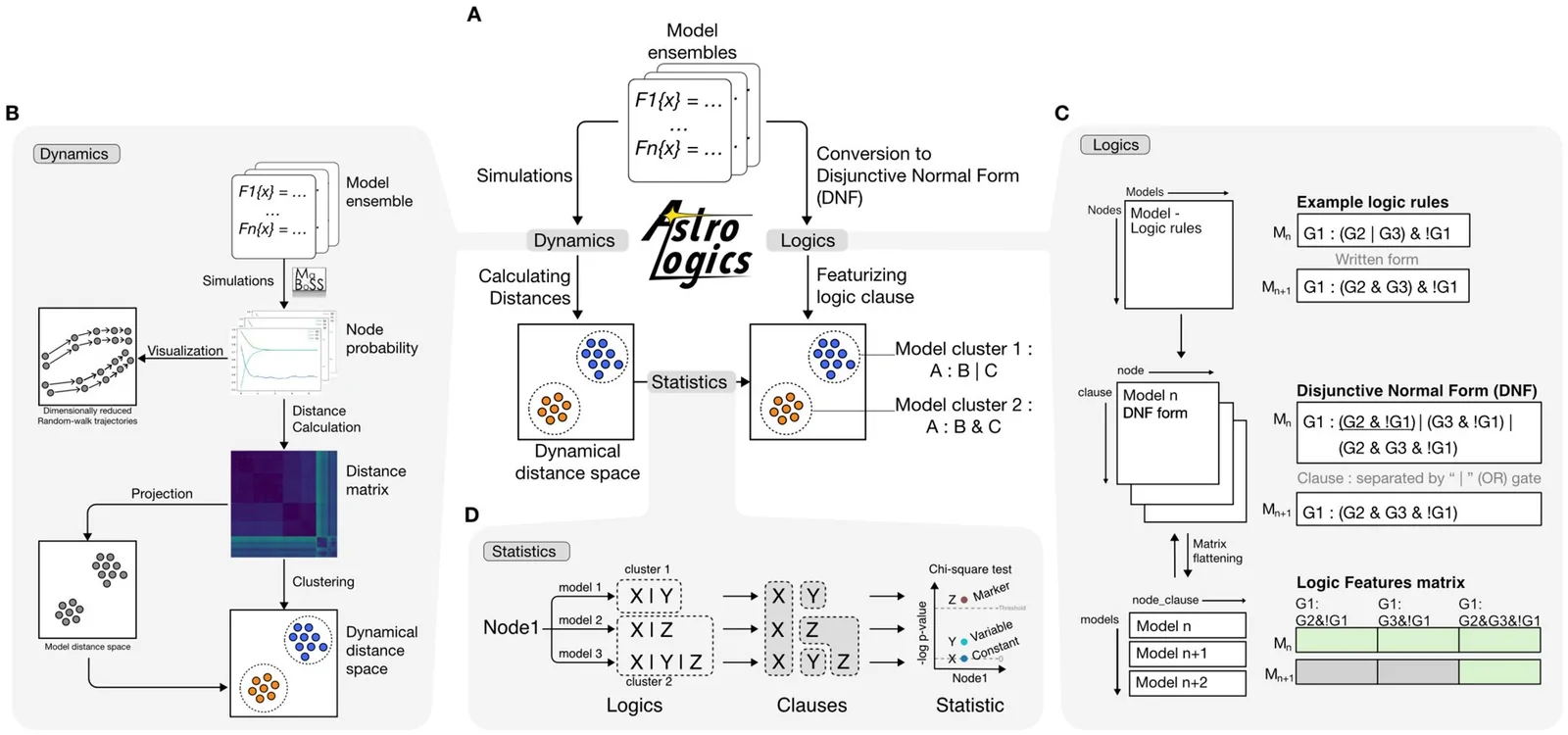
AstroLogics framework. (A) Workflow overview with three modules: *Dynamics* (clusters models by dynamics), *Logics* (featurizes logic rules), and *Statistics* (identifies key logic features). (B) *Dynamics module*. MaBoSS simulation produces a time series of node activation probabilities for each BN. This time series is then used to calculate pairwise distances, which serve as a basis to project and cluster trajectories. (C) *Logics module*. Left: BN logic rules matrix is converted into a logic features matrix. Right: example showing the processing of logic rules of node G in models *n* and n+1 into a DNF and then a binary logic features matrix (green = 1, grey = 0). (D) *Statistics module*. Per-node logic features are tested with chi-square against the Dynamics clusters and classified based on *P*-values into constant (all models), variable (non-significant), and marker (significant) features.

The AstroLogics framework aims to provide a tool for analyzing and identifying differences between Boolean models within the model ensemble. Each BN is characterized by two complementary properties. The distances between BNs are computed by comparing their simulated dynamics, and models are clustered into groups sharing similar dynamical behaviors. From the defined model clusters, differences in the logical equations between clusters are identified to characterize and interpret the regulatory rules underlying the observed separation. The AstroLogics workflow is illustrated in Fig. 1.

### 2.5 Dynamical features

STG calculation requires intensive computational power. Therefore, different methods have been developed to comprehend key STG features. Although attractors are key dynamical features, networks with identical attractors may exhibit different dynamical properties based on their STG structure. With MaBoSS, we specify initial parameters, including initial states, simulation time steps, and the number of sample counts. By default, our pipeline uses random initial states to perform a state space exploration, but initial states can be defined for detailed and context-specific analyses. These same initial-state distributions are assigned to all models in the ensemble. MaBoSS generates activation probabilities for each state, which can be converted into node activation probabilities and stored as a time-series matrix. This matrix can be used to visualize the dynamics of each node in the model. We also implemented a function that quantifies node variance at each simulation time step to help identify the most dynamically variable node (see Tutorials). From this matrix, AstroLogics calculates model differences using either terminal time point data (Euclidean distance) or whole trajectory data via Dynamic Time Warping (DTW) (Tavenard *et al*. 2020). We employ Non-metric Multidimensional Scaling (NMDS) (Kruskal 1964, Shepard 1962) to visualize the distance matrix in a 2-dimensional space, then perform k-means clustering to identify BN clusters with similar behavior, and project them onto the NMDS space (Fig. 1B).

### 2.6 Logic features

Logic rules describe how each node gets activated using logical operators, such as ∧ (AND), ∨ (OR), and ¬ (NOT). Since comparing these strings systematically is challenging, we convert them into a logic feature matrix. To obtain a comparable feature matrix, we convert each logic rule into a Disjunctive Normal Form (DNF). A DNF is a widely used, standardized format that avoids complex nesting and facilitates comparison (Crama and Hammer 2010). A DNF represents Boolean expressions using OR-connected “clauses,” where each clause combines variables using AND operators. For each Boolean network, we treat individual component clauses as features, creating a matrix where columns represent nodes, rows represent possible clauses, and values indicate the presence of each node-clause logic. We flatten this matrix into a vector, containing a combination of node_clause (referred to as a logic feature) for each model, then combine these vectors to create a final matrix where columns represent logic features, and rows represent individual models. We illustrated this processing in Fig. 1C. The left side of the panel shows the processing of the BN logic rules dataframe (top) into the logic features matrix (bottom). The right side of the panel illustrates the conversion of the logic rule of node G in the models *n* and n+1 into a DNF, and then into a binary logic feature matrix, where the color represents the value 1 (green) and 0 (grey). This conversion of logic rules into binary logic features enables their visualization and comparisons between BN groups using statistical methods.

### 2.7 Linking dynamical features with logical features

After clustering Boolean Networks (BNs) based on their dynamical properties, we sought to identify logic features accounting for cluster separations (Fig. 1D). To do so, we used Chi-square statistical tests on each logic feature to determine its association with specific model clusters. Based on *P*-values and a defined threshold, we categorized three logic feature groups: constant features (present in all models), variable features (high *P*-value), and marker features (low *P*-value, statistically significant between clusters). The logic feature distribution across models can be visualized in Manhattan plots, summary bar plots, or heatmaps. As described in the Results section, this approach enables the identification of key logic properties distinguishing different BN clusters and providing insights into which regulatory mechanisms are responsible for specific dynamical behaviors. These visualizations constitute the final output of the AstroLogics pipeline.

## 3 Results

To demonstrate the functionality and capability of our framework, we apply AstroLogics to three Boolean network ensembles, each chosen to highlight a distinct aspect of the pipeline. We first use a central nervous system (CNS) differentiation model (Qiu *et al*. 2017) to illustrate the core capabilities of AstroLogics. We then turn to the hematopoiesis model (Hérault *et al*. 2023) to introduce whole-trajectory clustering. Finally, we apply the framework to a cancer cell invasion model (Cohen *et al*. 2015), a more complex network that reflects realistic, application-scale use case. These models have been used to demonstrate BoNesis’ model inference functionality, with traceable constraints. This allows the interpretation of the clusters and marker logic features identified by AstroLogics.

### 3.1 Demonstration of AstroLogics functions

**Figure 2:**
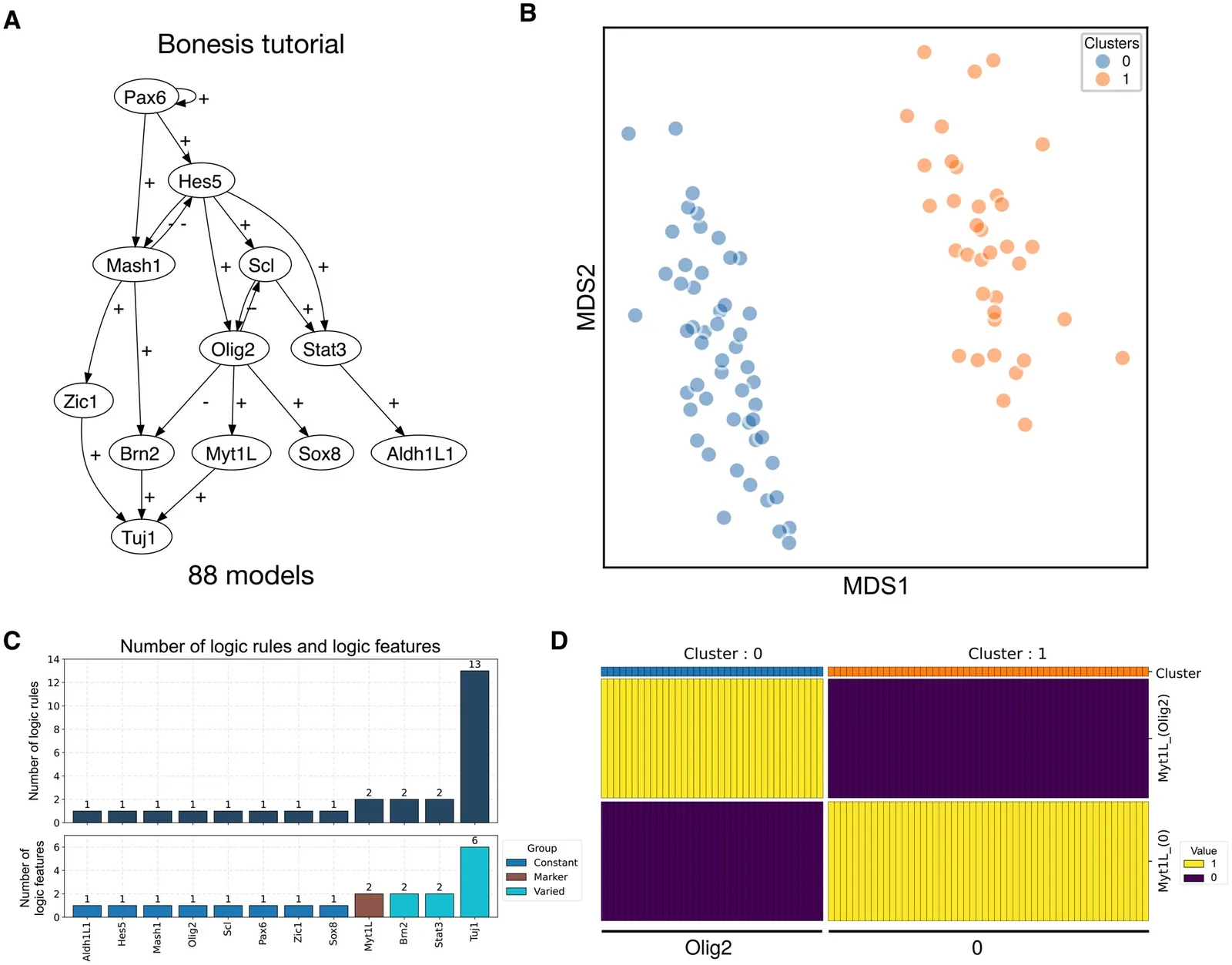
Application of the AstroLogics framework to a model for the differentiation of the central nervous system. (A) Regulatory graph displaying the components and regulatory interactions considered in the model. (B) Graph displaying distances between the models generated with BoNesis, based on the computation of stochastic asynchronous simulations with MaBoSS, and visualized using MDS. (C) Bar plots displaying the number of logic rules for each node (upper) and the number of logic features (bottom), with colors denoting logic feature classification (Dark blue = Marker feature, Brown = Constant feature, and light blue = Variable feature). (D) Binary logic-feature heatmap for Myt1L across models. Columns represent individual models and are annotated by cluster assignment (top color bar). Rows represent distinct Myt1L logic features. Heatmap values indicate feature presence in each model: yellow = 1 (present) and purple = 0 (absent).

To demonstrate the function of AstroLogics, we first study a model of the differentiation of the Central Nervous System (CNS) (Qiu *et al*. 2017) (Fig. 2A), which has been presented in a tutorial network for BoNesis (Chevalier *et al.* 2024). We generated the ensemble of 88 models (cf. https://bnediction.github.io/bonesis/tutorials/tour.html) and applied our approach. We first focused on the attractors and their reachability. We found that within the model ensemble, BNs can be separated into two major groups according to the set of reachable attractors (Fig. 2B). We then asked what key logic features distinguish these two groups of BNs. Following the logic comparison pipeline of AstroLogics (see Section “Materials and methods”), we were able to identify three major groups of logic features: (i) constant features, which are logic features that are included in all BNs and can be seen as the backbone of the model; (ii) variable features, which are logic features that may vary between BNs but are not statistically significant between clusters of BNs; and (iii) marker features, which are logic features that are statistically significant between two clusters of models. To identify the nodes of interest that contribute to these marker features, we generated two bar plots, one showing the number of possible logic rules for each node (corresponding to all the possible logic rules) and the other showing the number of logic features that contribute to all possible logic rules in each node (Fig. 2C).

This analysis reveals that eight nodes contain only a single logic rule each (Adh1L1, Hes5, Mash1, Olig2, Scl, Pax6, Zic1, and Sox8). We found that the four nodes Myt1L, Brn2, Stat3, and Tuj1 have multiple logic rules, leading to dynamic differences across models. However, among all these nodes, we found that only Myt1L contains logic features that are categorized as marker features. Hence, we conclude that Myt1L is a key node underlying differences in the dynamical behavior of the two groups of models. Despite the high number of possible logic rules for Tuj1 in the model ensemble, this node doesn’t drive the separation between model clusters. This suggests that node uncertainty may not predict model separation, and that simulation-based clustering better captures this distinction. We then displayed the different Myt1L logic features (Fig. 2D). From this visualization, we found that in cluster 0, Myt1L is regulated by Olig2, while in cluster 1, Myt1L is deactivated 0. Retracing the model inference process, we found that the underlying constraints specify node values at the initial state (init) and at several transition and stable states (tM, fT, tO, fMS, tS, fA). Myt1L is constrained only in the zero (unreachable states with all components inactivated) and initial (init) state and is left unspecified in all transition and stable states (see BoNesis tutorial), allowing BoNesis to assign any consistent logical rule to it. Therefore, the observed Myt1L-driven cluster separation reflects loose model inference constraints. This finding illustrates how AstroLogics can guide subsequent model refinements.

### 3.2 Comparison of trajectory clustering and attractor analysis approaches

Inspired by the AEON approach (Beneš *et al.* 2020), which exploits long-term behavior of BN to characterize BNs into groups, we used the attractor calculation function provided by the package boolsim (Garg *et al*. 2008) to calculate a set of the model’s attractors. We then clustered the models based on the calculated sets of attractors. We compared this attractor-based clustering with our simulation-based clustering using 19 selected networks from Kadelka and colleagues (Kadelka *et al*. 2024). For each network, we generated 1000 random BN model ensembles using BoNesis. We calculated attractors for each model and clustered them. In parallel, we performed MaBoSS simulations on all models, obtained node activation probabilities at the terminal time point, and calculated Euclidean distances between models. Since the optimal number of clusters for simulation-based clustering was unknown, we performed *k*-means clustering with varying cluster numbers ranging from 1 to *n* (where *n* is the number of attractor-based clusters). We then calculated the Adjusted Rand Index (ARI) to compare the resulting clusters. Finally, we selected the maximum ARI for each network, as using n clusters in simulation-based clustering does not guarantee maximum agreement with attractor-based results.

The results for the 19 selected networks are recapitulated in Table 1 with the characteristics of each model, such as the number of nodes and interactions, the number of models generated with Bonesis, the number of clusters obtained with the attractor identification approach, and the ARI-score from the terminal simulation time points. These results reveal a good agreement with the attractor-based clustering approach. Indeed, 16 out of 19 models led to ARI scores >0.85, while only three networks led to relatively low ARI scores (<0.45). We further investigated this issue in Supplementary Analysis 1, available as supplementary data at *Bioinformatics* online.

**Table 1:** Correspondences between attractors and simulation-based clustering.

Project name	Nodes	Interactions	Num model	Num attractors groups	Max ARI (endpoint)
Body Segmentation in Drosophila 2013_23520449	14	12	1000	86	1
Death Receptor Signaling_20221256	25	45	1000	16	1
FGF pathway of Drosophila Signaling Pathways_23868318	14	24	1000	570	1
HH Pathway of Drosophila Signaling Pathways_23868318	11	30	1000	1000	1
Mammalian Cell Cycle_19118495	19	44	1000	7	1
Processing of Spz Network from the Drosophila Signaling Pathway_23868318	18	20	1000	152	1
Toll Pathway of Drosophila Signaling Pathway_23868318	9	11	16	6	1
VEGF Pathway of Drosophila Signaling Pathway_23868318	10	18	1000	390	1
Regulation of the L-arabinose operon of Escherichia coli_28639170	9	16	1000	130	0.990976
SKBR3 Breast Cell Line Long-term ErbB Network_24970389	21	52	1000	41	0.977437
Cardiac development_23056457	14	24	1000	6	0.977327
HCC1954 Breast Cell Line Short-term ErbB Network_24970389	11	33	1000	304	0.962528
Lac Operon_21563979	10	22	1000	61	0.961812
SKBR3 Breast Cell Line Short-term ErbB Network_24970389	11	29	1000	268	0.931461
B cell differentiation_26751566	17	38	1000	287	0.907193
BT474 Breast Cell Line Long-term ErbB Network_24970389	21	41	1000	249	0.887925
Human Gonadal Sex Determination_26573569	19	76	1000	6	0.442307
HCC1954 Breast Cell Line Long-term ErbB Network_24970389	21	42	1000	542	0.362385
Cortical Area Development_20862356	5	11	1000	5	0.138381

We emphasized that AstroLogics classifies Boolean models primarily based on their dynamical behavior, such as the sets of reachable attractors. To assess the added value of this dynamics-based strategy relative to a simpler, structure-based alternative, we benchmarked AstroLogics against distances computed directly from structural edge features. Using the Death Receptor Signaling_20221256 network from (Kadelka *et al*. 2024), we compared (i) clustering derived from the simulation-based distance matrix with (ii) clustering derived from structural edge features (Supplementary Analysis 2, available as supplementary data at *Bioinformatics* online). The result confirmed that the dynamics-based representation captures information that is not recoverable from network structure alone, supporting the use of simulated dynamics as the primary input to AstroLogics.

### 3.3 Using transient dynamics of BNs to cluster models

MaBoSS simulations output the probabilities for node activations at successive time points, thereby enabling the analysis of transient dynamics. While attractors are valuable for identifying long-term system behaviors corresponding to phenotypes or cell fates, they offer only a limited view of the model dynamics. The state transition path leading to an attractor often contains crucial biological information, such as transient gene activation and signaling cascades. This is particularly important in development and in immune responses, where the timing and order of activation play critical roles. Furthermore, two models with identical attractors may follow significantly different paths, which can impact the interpretability and the delineation of intervention strategies.

We applied the Dynamic Time Warping (DTW) algorithm (Sakoe and Chiba 1978), implemented in the tslearn package (Tavenard *et al*. 2020), to compute distances between models, considering the whole simulation trajectories rather than focusing only on the terminal time point. This resulted in a distance matrix similar to the Euclidean distance matrix, which can be used for model visualization in the MDS latent space, and/or perform model clustering.

To verify that considering the whole trajectories rather than terminal time points alone does not reduce the efficacy of categorizing models, we performed simulations, computed BN distances using the whole simulation trajectories, clustered BNs, and measured the agreement of the resulting clusters with the attractor-based clustering approach. We compared ARI scores between whole-trajectory and terminal time point clustering, as shown in Fig. S2, available as supplementary data at *Bioinformatics* online. We confirmed that the classification efficacy remained equivalent across both approaches, demonstrating that the use of whole trajectories does not compromise the accuracy of attractor-based model categorization.

**Figure 3:**
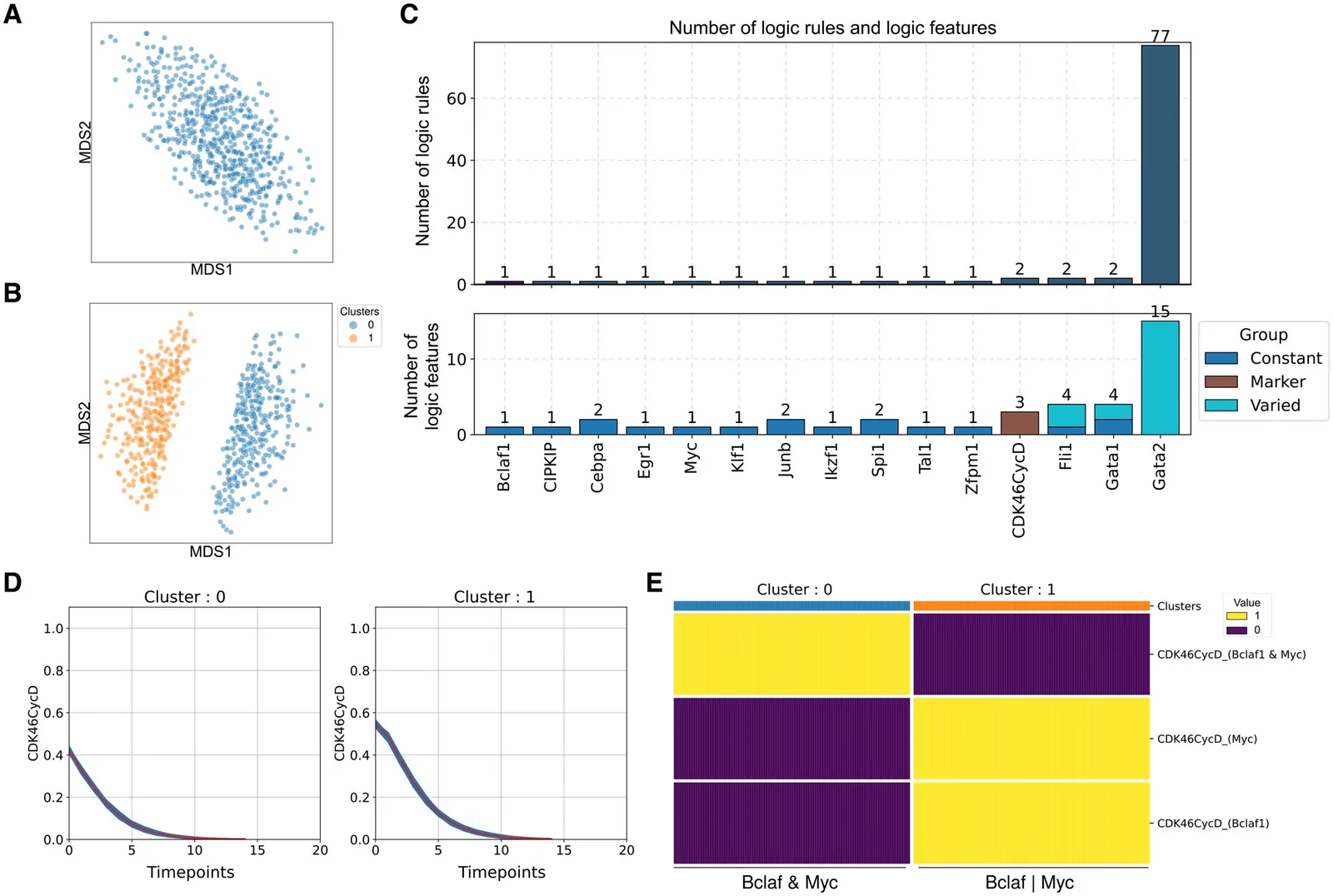
Analysis of transient dynamics in Boolean network models. MDS projection of BNs distances from the published hematopoiesis model ensemble, where distances are calculated from (A) the terminal time point or (B) the whole trajectories. (C) Bar plots displaying the number of logic rules for each node (upper) and the number of logic features (bottom), with colors denoting logic feature classification (Dark blue = Marker feature, Brown = Constant feature, and light blue = Variable feature). (D) Visualization of CDK46CycD node activation probabilities over the simulated time. (E) Logic features of node CDK6CycD. Each row represents the CDK6CycD logic feature, while each column represents a model in the model ensemble, with column color indicating the model cluster.

We then focused on the biological insights that could be gained by exploring transient events. For that purpose, we relied on a previously published BN model ensemble from a study on hematopoiesis (Hérault *et al*. 2023). The ensemble consists of 616 models that share the same single attractor. We carried out MaBoSS simulations across all 616 models and calculated inter-model distances using both terminal time point results and whole simulation trajectories. Distances derived from the terminal time point failed to produce clear clustering within the ensemble, which is in accordance with the identification of a single attractor in the initial publication (Fig. 3A). In contrast, trajectory-based distances revealed a clear division of the Boolean networks into two distinct groups (Fig. 3B). The separation is due to the logic features of CDK46CycD (shown in Fig. 3C). The dynamics of this Cyclin/CDK complex in both clusters reveal subtle early-stage differences that eventually converged to zero at steady state (Fig. 3D). These differences are clearly evident in the distinct logic rules within each cluster. In cluster 0, all models share the logic: Bclaf1 ∧ Myc, making both Bclaf1 and Myc necessary for CDK4CycD activation, while for cluster 1 models, one or the other input is enough to activate the complex. This result may reflect plausible cell-to-cell variability in G1 duration (shorter vs. longer CDK46CycD activation windows) or under-constrained inference. Based on this result and depending on the context, an additional constraint may be added for a more refined definition of the ensemble that would reduce the number of plausible models. This case study illustrates how AstroLogics can help understand the structure of model ensembles, potentially enabling their refinement.

### 3.4 Case study: cancer invasion model

**Figure 4:**
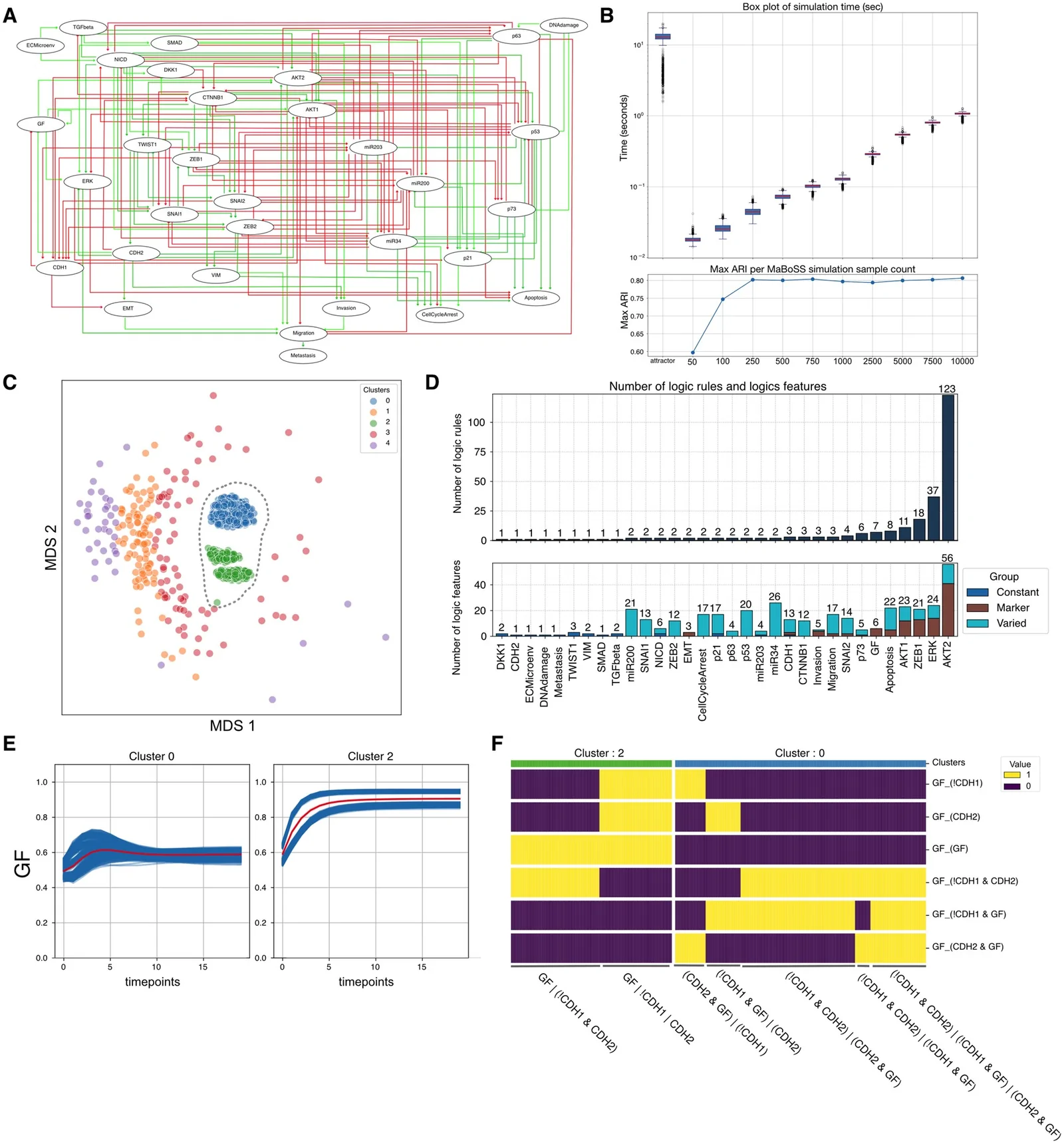
Application of AstroLogics to a complex cancer invasion model. (A) Network diagram of the cancer invasion model (Cohen *et al*. 2015) showing key nodes and interactions involved in the EMT process of breast cancer. (B) Effect of MaBoSS simulation sample count on computation time and clustering agreement. (Top) Box plots of computational time (runtime in seconds) required for attractor calculation (through boolsim) and MaBoSS simulation for different sample counts. (Bottom) Line plots of maximum ARI showing the agreement between model clusters obtained with the attractor-based approach and the MaBoSS simulation-based approach, considering different sample counts. (C) MDS projection of the 1024 models generated following Chevalier et al.’s methodology and constraints. Five model clusters can be distinguished, denoted by different colors. A dashed line delineates clusters 0 and 2. (D) Plots showing the number of possible logic rules (upper) and logic features (lower) for each node across models of clusters 0 and 2. The colors in the stacked bar plot represent the number of logic features categorized as constant (dark blue), varied (light blue), and marker (brown). (E) Visualization of node activation probabilities for the GF node in clusters 0 and 2, highlighting substantial differences in GF activity patterns. (F) Heatmap showing the different logic features of node GF. Each row represents a GF logic feature, while each column represents a model in the model ensemble.

Finally, we applied our approach to a Boolean model of cancer cell invasion (Cohen *et al*. 2015) (Fig. 4A). We followed the BN inference method detailed in (Chevalier *et al.* 2020), which generated a model ensemble of 1024 models.

One of the difficulties in analyzing this complex model comes from the high number of reachable attractors, which requires extensive computational resources. In our framework, we circumvent this issue by estimating the computation time with MaBoSS. We compared the computational time required for the exact calculation of the attractors using boolsim with MaBoSS simulation times (ranging from 50 to 10 000 sample counts), varying the number of simulated trajectories. For this whole sample counts range, we showed that simulations with MaBoSS offered a significant time advantage for calculating dynamical properties. Moreover, ARI scores across sample counts indicate that already with 250 samples, the clustering shows strong agreement with the reference grouping (ARI approx. 0.81, Fig. 4B). To control the impact of stochasticity in MaBoSS simulations, we performed a convergence analysis considering different sample counts (see Supplementary Analysis 3, available as supplementary data at *Bioinformatics* online). A sample count of about 2000 offers a good trade-off between convergence and computational cost, and we thus used this sample count for the subsequent analyses.

We then computed model distances obtained with MaBoSS simulations and projected them onto the MDS latent space with k-means clustering. Five distinct model clusters were identified (Fig. 4C). Focusing on clusters 0 and 1, we found that these clusters have similar logic feature profiles (Fig. S3, available as supplementary data at *Bioinformatics* online). The similarities between clusters consist of conserved regulatory logic motifs with only a few nodes identified as marker nodes (Fig. 4D). To investigate these differences further, we selected the nodes showing the most dynamic variation across all models: CDH1, EMT, Invasion, Apoptosis, Migration, SNAI2, GF, AKT1, AKT2, ERK, and ZEB1. Among these nodes, GF (growth factors) shows a notable difference in activity between clusters 0 and 2 (Fig. 4E, Fig. S4, available as supplementary data at *Bioinformatics* online). Examining the logical features within each cluster reveals the presence or absence of auto-activation of the GF node (Fig. 4F).

Of note, all figures and plots presented in the examples can be replicated with the corresponding scripts along with detailed tutorials on the GitHub page: https://github.com/sysbio-curie/AstroLogics.

## 4 Discussion

The field of BN inference has advanced significantly over the past decades. Several recent model inference methods use machine-learning optimization pipelines (Li *et al.* 2023, Zhang *et al*. 2024, 2025), where each target gene is treated as a separate learning task, scoring candidate rules from time-series data, and then selecting a single best Boolean network via model selection. This workflow is easier to implement under the synchronous update semantics assumed in these studies. By contrast, many Answer Set Programming(ASP)-based inference frameworks considered here target asynchronous or most-permissive updates, which are usually considered as more relevant from a biological point of view (Liang *et al*. 1998, Lähdesmäki *et al.* 2003, Ostrowski *et al*. 2016, Chevalier *et al*. 2019). Under these semantics, the induced dynamics are non-linear and harder to encode in standard objective functions. Thus, an inference attempt often outputs relatively large model ensembles. Analyzing these model ensembles provides valuable insights into the key inferences and highlights similarities among models within the ensemble. However, proper and efficient tools to compare models within the solution space are still lacking. In this study, we aimed to address this gap with the development of AstroLogics. AstroLogics is a comprehensive framework designed for analyzing BNs within the model ensembles that arise from BN inference methods. It evaluates BN models through two complementary approaches, focusing on the analysis of key dynamical properties and on a comparative analysis of the logic rules. Overall, AstroLogics is an exploratory analysis framework that generates hypotheses from inferred BN ensembles. These hypotheses may reflect either biological mechanisms or missing information in the inference constraints, which can then guide experimental design or further refinement of the model inference method.

Our dynamical model analysis considers both the long-term behavior of the BN and its transient dynamics. Existing classification methods, such as AEON and BNclassifier (Beneš *et al.* 2020, 2024), primarily classify BNs based on their attractors. These attractor-based clustering approaches are adapted to analyze Parameterized Boolean networks (PBN), which involve incompletely specified Boolean functions (in .aeon file format), while our framework handles heterogeneous ensembles where both structure and update logic can differ across models (multiple .bnet files). The BNclassifier investigate how changes in logical parameters affect the attractor landscape. They rely on symbolic algorithms to compute and categorize attractors following three coarse properties: stability (single state), oscillations (periodic cycle), and disorder (chaotic behavior). Meanwhile, the AstroLogics framework clusters fully specified models by simulation-based dynamical similarity and uses logic features to explain cluster differences. Hence, the two frameworks should be considered as complementary.

MaBoSS simulation provides flexibility through tunable transition rates ($R^{up}$ and $R^{\mathrm{down}}$) (see Supplementary Material, available as supplementary data at *Bioinformatics* online). In the current framework, distances are computed from time series assuming equal activation and inactivation probabilities across all nodes, without prioritizing any subset. In specific use cases, however, introducing node-level weights could be valuable. For instance, in gene regulatory networks (GRNs), which comprise both transcription factors (TFs) and their target genes, weighting could be used to prioritize TF activation over target gene activation. We plan to investigate such application-specific extensions in future work. However, the use of MaBoSS also restricts us to its continuous-time asynchronous update scheme. Therefore, the exploration of synchronous or hybrid update modes is not possible. Moreover, this precludes the application to probabilistic Boolean networks (PBNs) with weighted transitions. In detail, a PBN requires a set of candidate predictor functions ($F_{i}=\{f_{i}^{1},f_{i}^{2},\ldots ,f_{i}^{n}\}$) for each variable ($x_{n}$), together with a probability vector ($c_{i}=\{c_{i}^{1},c_{i}^{2},\ldots ,c_{i}^{n}\}$), where $\sum_{n}c_{i}^{n}=1$ (Shmulevich *et al*. 2002). In AstroLogics, the logic-feature extraction assumes Boolean update rules whose DNF representation is well-defined. A probabilistic transition kernel would therefore require an extension of the feature representation to incorporate both the predictor functions and the associated probability vector.

Despite this discrepancy, our simulation approach reduces computation time when analyzing models with multiple, complex attractor states (Fig. 4B). We therefore present this method as an efficient approximation for exploring and comparing model dynamics. However, modern single-cell gene regulatory network studies have accelerated the growth in network size, which can exceed hundreds of nodes (Badia-I Mompel *et al*. 2023). We tested the method on a model with over 300 nodes and 250 interactions (Signaling in Macrophage Activation_18433497). As expected, the simulation time is much longer (about 40 seconds per model) than for smaller models. Nevertheless, the method remains applicable at this scale (a script for this analysis can be found in Results_large_model on the GitHub repository). To address the increased runtime, a recent GPU implementation of MaBoSS (Šmelko *et al.* 2024) could potentially scale simulations by more than 100-fold (based on real-world example models), enabling the simulation of much larger and more complex models.

The comparison between our simulation-based and the attractor-based approaches demonstrated global agreement across most models, but discrepancies were observed in some networks. We demonstrated, in Supplementary Analysis 1, available as supplementary data at *Bioinformatics* online, that these discrepancies arise from the reachability of attractors. We found that models that differ in low-reachability attractors exhibit only minor differences in distance, resulting in minimal distances in the MDS space. Despite this discrepancy, we believe that accounting for the probability of reaching the attractors will better reflect the dynamics of the BNs.

While long-term BN behavior is important, transient dynamics can also play key roles in determining the timing and order of activation of BN components involved in cell fate decisions. Even when BNs share the same attractors, different transient dynamics can shape distinct phenotypic timing and variability. These transient dynamics can reveal key control points and time windows where perturbations can be applied to redirect BN behavior. Therefore, incorporating the transient dynamics discriminates models that are indistinguishable by attractors alone, clarifies signal propagation, and reveals actionable control points under realistic time-dependent constraints.

The AstroLogics pipeline relies on the DNF to convert logic rules to features. However, the DNF representation of a complex logical formula is not unique, which could complicate their comparisons (see Supplementary Information, available as supplementary data at *Bioinformatics* online for an example). The use of the so-called “completed DNF” (also known as Blake’s Canonical Form) (Blake 1938) constitutes an interesting alternative. Complete DNFs (CDNFs) are defined as the disjunction of all prime implicants, themselves defined as minimal partial assignments that make a Boolean function true. The use of CDNF could potentially help identify redundant BNs within model ensembles (See Supplementary Information, available as supplementary data at *Bioinformatics* online for more detail). However, our pipeline currently doesn’t support the conversion to CDNF.

The AstroLogics framework integrates a method to compare the long-term behavior of BNs and cluster them using boolsim. In recent years, new methods to identify attractors have arisen, including symbolic, structure, or decomposition-based approaches (reviewed in (Trinh *et al*. 2022)), enabling the analysis of larger models, with lower computational time. As the field continues to evolve, it would be interesting to explore and implement some of these approaches in future versions of our package. These enhancements would further strengthen the capability of AstroLogics to analyze complex biological networks.

Recent advances in Boolean network analysis have expanded our understanding of network dynamics beyond traditional attractor identification. The concept of trap spaces, i.e. cubic subspaces from which trajectories cannot escape, has emerged as a powerful analytical tool (Klarner *et al.* 2014). A trapspace can be represented by a stable motif, revealing the frozen components, i.e. the components keeping the same value, 0 or 1, independently of the other components, at or near complex attractors (Zañudo and Albert 2013). The stable motifs limit the accessible state space, channeling dynamics toward specific attractors. Recently, several groups proposed global representations of the dynamics, including long-term behavior in terms of acyclic graphs, called the Commitment Diagram (Klarner *et al.* 2020) or the Succession Diagram (Rozum *et al.* 2021).

To facilitate the incorporation of novel dynamical analysis methods, we have designed our framework in a modular fashion, supporting different model dynamics calculation strategies, including attractor analysis and succession diagrams, as implemented by pystablemotifs (Rozum *et al*. 2022). Our framework further fosters interoperability by adhering to the standardized CoLoMoTo environment. Taking advantage of the tools already integrated in the environment, including minibn for model import and boolsim for attractor calculation, AstroLogics ensures seamless integration with existing Boolean modeling workflows. Finally, the combination of a modular design with CoLoMoTo packages enhances cross-platform compatibility and improves reproducibility across different computational environments (Noël *et al*. 2025).

## Supplementary Material

btag555_Supplementary_Data

## Data Availability

The developed AstroLogics package is available on GitHub https://github.com/sysbio-curie/AstroLogics, as well as datasets, models, and scripts to replicate each experiment and figure shown in this work.
