# Supplementary material for: AstroLogics: a simulation-based framework for the analysis of boolean model ensembles
Source: Bioinformatics. 2026 Jul 25;42(8):btag555. doi: 10.1093/bioinformatics/btag555 (PMC13472726; doi:10.1093/bioinformatics/btag555)
Supplement: Supplementary file 1 [file btag555_supplementary_data.pdf]

Supplementary Materials - AstroLogics: A simulation-based framework for the analysis of Boolean model ensembles

Pankaew et al.

July 21, 2026

Contents

1 Supplementary Information **2**
1.1 Boolean network . . . 2
1.1.1 Definitions . . . 2
1.1.2 Dynamical properties . . . 2
1.1.3 Updating Schemes . . . 2
1.1.4 State Transition Graph and Attractors . . . 2
1.2 MaBoSS simulation . . . 3
1.2.1 Calculating states activation probability . . . 3
1.2.2 Transition rates . . . 3
1.2.3 Calculating node activation probability . . . 3
1.3 Measuring distance between BN's dynamics . . . 4
1.4 Non-uniqueness of DNF . . . 4
1.5 cDNF and its problem . . . 5
1.5.1 DNF blow-up . . . 5

2 Supplementary Figures **6**
2.1 Supplementary Figure S1 . . . 6
2.2 Supplementary Figure S2 . . . 7
2.3 Supplementary Figure S3 . . . 8
2.4 Supplementary Figure S4 . . . 9

3 Supplementary Analysis **10**
3.1 Supplementary Analysis 1: Low ARI Scores . . . 10
3.1.1 Analytical pipeline . . . 10
3.1.2 Results . . . 10
3.1.3 Interpretation . . . 10
3.2 Supplementary Analysis 2: Comparison with Edge-Based Methods . . . 12
3.2.1 Analytical pipeline . . . 12
3.2.2 Results . . . 12
3.2.3 Interpretation . . . 12
3.3 Supplementary Analysis 3: Convergence Analysis . . . 13
3.3.1 Analytical pipeline . . . 13
3.3.2 Results . . . 14

1 Supplementary Information

1.1 Boolean network

1.1.1 Definitions

A Boolean network is define by a regulatory graph (V, E) endowed with a set of dynamical parameter (K). This regulatory graph is a signed directed graph with the following definition:

- $V = v_1, v_2, v_3, \ldots, v_n$ a set of n nodes that represent the n components of the biological network (ex. genes, proteins, complexes, etc.).
- E a set of signed directed edges representing the interactions between components, with a sign + for activation, and - for inhibition.

The regulatory graph shows static interaction between selected biological components. To represent the system's dynamics from this interaction, we attach a variable to each node and parameterize the regulatory graph as follows: Each node v_i is associated with a discrete variable D_i in $0, 1$, and K_i is a set of logical functions defining each node's target state based on the states of its regulators. More precisely, the function K_i can be written as a logical rule for a given node for instance, K_n for v_n, so that $v_n = 1$ when $(v_i \wedge v_j) \vee \neg v_k$.

1.1.2 Dynamical properties

After constructing the model, we examine its dynamics through simulation. Understanding these dynamics relies on two key concepts: **states** and **transitions**. Because the dynamics are encoded by the logical parameters K_i, it is useful to unfold them to generate the possible trajectories of the system. A state of the system is a vector $s = (s_1, s_2, \ldots, s_n)$, where n is the number of components and s_i is the state of component v_i. In a Boolean model, the number of possible states is 2^n, but the logical rules constrain which states can transition to one another.

1.1.3 Updating Schemes

The qualitative abstraction process also encompasses time. In Boolean models, time is not an absolute unit. Instead, it reflects the sequential nature of state transitions in a discrete-event dynamical system. The update scheme is therefore crucial, and two common schemes are synchronous and asynchronous updating. In this example, we define a transition between two states x and y, where $x, y \in S$. With synchronous updating, **all components** are updated simultaneously after each transition, yielding deterministic dynamics:

$$x \to y \iff \forall i = 1, \ldots, n,\ y_i = K_i(x).$$

With asynchronous updating, components are updated **one at a time**, which can reveal trajectories hidden by synchronous updating:

$$x \to y \iff \exists!\, i \text{ s.t. } x_i \neq y_i,\ y_i = K_i(x), \text{ and } \forall j \neq i,\ y_j = x_j$$

Asynchronous updating demands more computational power, with each state having up to n outgoing transitions, yielding at most $n \cdot 2^n$ transitions overall. While synchronous updating requires less computation, simultaneously updating all components is biologically unrealistic (Fauré et al., 2006) and often fails to capture differences in the time scale of individual updates. In this work, we use asynchronous dynamics for more realistic trajectories.

1.1.4 State Transition Graph and Attractors

This dynamic appears in the state transition graph (STG) representation, where nodes represent states and directed edges show transitions. Based on the State transition graph, multiple features can be extracted from this network, including basins of attraction, reachability, and others. However, one of

the key prominent features in the STG is the terminal states. With finite states, simulation leads to terminal state(s) called attractor(s). Attractors, defined as state sets with no escape transitions, come in two types:

- Single attractors (stable states): States where component values remain constant
- Cyclic attractors: Sets of mutually reachable states with no outward transitions

These attractors correspond to biological phenomena, for example, single attractors often represent cell differentiation states (Collombet et al., 2017), while cyclic attractors typically represent periodic behaviors like cell cycles or circadian rhythms (Traynard et al., 2016; Hassan et al., 2018).

1.2 MaBoSS simulation

1.2.1 Calculating states activation probability

The AstroLogics framework uses MaBoSS to simulate each BN in the model ensemble. MaBoSS applies the Gillespie (Kinetic Monte-Carlo) algorithm to sample stochastic trajectories through the Boolean state space and compiles them into state probability distributions at each discrete time step. From a current network state S, it computes the total outgoing rate as:

$$\rho_{total} = \sum_{S'} \rho_{S \to S'}$$

Each single-node flip i is weighted by its transition rate (activation or inhibition) rate $R_i^{up/down}(S)$, draws a waiting time (δt), where:

$$\delta t = -\frac{ln(u)}{\rho_{total}}$$

From this waiting time, the next state is selected by the probability of $\rho_{S \to S'}/\rho_{total}$. Repeating this generates N stochastic trajectories $\hat{S}(t)$. Time is then discretized into windows of width δt. For each trajectory, the fraction of time spent in each network state within window τ is measured, and averaging across the N trajectories yields the empirical state probability as follows:

$$\hat{P}[s(\tau) = S]$$

1.2.2 Transition rates

The transition rates of MaBoSS replace the unweighted transitions of standard asynchronous Boolean updating and set the biological time scale of every node flip. The transition rate of each node contains $R_i^{up}(S)$ or $R_i^{down}(S)$, which can be made state-dependent. This allows the modeler to encode different speeds for different inputs, slow buffering effects, or multi-level variables without changing the logic rules. The relative levels of these rates control the trajectories sampled, where the fast rates dominate the choice of the next transition.

1.2.3 Calculating node activation probability

As MaBoSS samples stochastic trajectories through the Boolean state space and compiles them into state probability distributions at each discrete time step. Formally, a network state S encodes the Boolean value of every node ($S_i \in \{0, 1\}$). The node activation probability for node i at time τ is obtained by summing over all network states in which node i is active:

$$P[S_i(\tau) = 1] = \sum_{S\,:\,S_i=1} P[s(\tau) = S]$$

This amounts to summing the probabilities of all 2^n (where n is the number of nodes in the model) network states that have $S_i = 1$. Therefore, in MaBoSS notation, expressions such as Prob[CycA=1]

or Prob[p53=1**...] represent the probability aggregated over all states consistent with a given node being ON, regardless of the values of other nodes. From this notion, each model is then reduced to a single, deterministic multivariate time series of node activation probabilities (one scalar per node per time step). This serves as the comparable representation for inter-model distance computation.

1.3 Measuring distance between BN's dynamics

In AstroLogics, each BN is simulated with MaBoSS, resulting in a multivariate time series of node activation probabilities over time. The most direct option for such comparison is through **Euclidean distance**, which compares two trajectories point-by-point.

$$d_{Euc}(\mathbf{X}, \mathbf{Y}) = \sqrt{\sum_{t=1}^{T} \|x_t - y_t\|^2}$$

In AstroLogics, this method is used to calculate distances between models when using the end time point simulation. However, for the whole trajectories comparison, we decided to use **Dynamic Time Warping (DTW)** to measure the distance between multivariate time series of node activation probabilities over time. A small DTW distance indicates similar temporal patterns and likely convergence toward a comparable set of attractors. A large distance reflects divergent dynamics that may correspond to different fates or responses. The DTW calculates the distance between models using the following approach:

$$d_{DTW}(\mathbf{X}, \mathbf{Y}) = \min_{\pi} \sqrt{\sum_{(i,j)\in\pi} \|x_i - y_j\|^2}$$

where (π) is a warping path matching time points across the two series. This helps align trajectories along the time axis, compared to Euclidean distance, which compares trajectories at the exact time point. This can be relevant for BN simulations, where models can exhibit similar qualitative dynamics but reach them at different rates.

1.4 Non-uniqueness of DNF

Despite the standard form of DNF, the DNF is not a unique representation of the logic rules. Because DNF is not a canonical representation, logically equivalent but formally distinct rules can be generated, leading to redundant models within the ensemble. This redundancy directly affects the logic feature matrix: two models harboring equivalent Boolean functions may be assigned different feature vectors, potentially distorting downstream statistical analyses. To illustrate this point, we consider two logical functions $f_1 = A \vee (B \wedge C)$ and $f_2 = (A \wedge B) \vee (A \wedge \neg B) \vee (B \wedge C)$. These produce identical truth tables, yet their DNF decompositions yield different clause sets. When converted to logic features, f_1 and f_2 are encoded by the four candidate clause features: A, $(A \wedge B)$, $(A \wedge \neg B)$, and $(B \wedge C)$.

- The function f_1 activates only the single-literal feature A and the conjunction $(B \wedge C)$. This results in a vector of $(1, 0, 0, 1)$
- The function f_2 activates the two conjunctions $(A \wedge B)$ and $(A \wedge \neg B)$ together with $(B \wedge C)$, but does not include the bare literal A. This resulting the vector $(0, 1, 1, 1)$

These features showed a difference that would incorrectly flag these models as different. So, we mentioned the use of the complete DNF (cDNF) as a solution to this limitation. Converting all logical rules to their cDNF before feature extraction would guarantee a canonical representation, allowing users to identify and merge such equivalent functions within the model ensemble before proceeding with downstream simulations.

1.5 cDNF and its problem

A **complete DNF (cDNF)** is a DNF written as a disjunction of minterms, where each minterm is a conjunction that includes every regulator exactly once (either as x or $\neg x$). Concretely, if a node has regulators (A, B, C), then a cDNF clause looks like $(A \wedge \neg B \wedge C)$, and the full cDNF is the OR of all such minterms for which the function evaluates to 1. This construction is unique for a fixed variable ordering because it is determined solely by the truth table: one minterm per satisfying assignment.

1.5.1 DNF blow-up

A principal caveat of the featurization pipeline is **DNF blow-up**, where converting large formulas to an exact DNF representation can grow exponentially with the number of regulators. Another way to see why this blow-up happens is to consider that distributing AND over OR can multiply the number of terms. For example, a rule of the form $(A_1 \vee A_2 \vee \cdots \vee A_k) \wedge (B_1 \vee B_2 \vee \cdots \vee B_k)$ expands in DNF to k^2 conjunctions ($A_i \wedge B_j$ for all pairs). With r such OR-blocks of size k, the number of clauses becomes k^r. In Boolean networks, high in-degree nodes often yield nested OR/AND structure, so exact DNF can quickly generate thousands to millions of clauses, increasing memory use and making feature matrices extremely high-dimensional and sparse.

In practice, this remains manageable for most biological rules, which typically have a limited in-degree, but several safeguards can be applied when needed, such as capping the maximum clause size, limiting the total number of clauses retained per rule, or resorting to alternative canonical forms such as BDD-based enumeration.

References

S. Collombet, C. van Oevelen, J. L. Sardina Ortega, W. Abou-Jaoudé, B. Di Stefano, M. Thomas-Chollier, T. Graf, and D. Thieffry. Logical modeling of lymphoid and myeloid cell specification and transdifferentiation. *Proceedings of the National Academy of Sciences*, 114(23):5792–5799, June 2017. doi: 10.1073/pnas.1610622114.

A. Fauré, A. Naldi, C. Chaouiya, and D. Thieffry. Dynamical analysis of a generic Boolean model for the control of the mammalian cell cycle. *Bioinformatics*, 22(14):e124–e131, July 2006. ISSN 1367-4803. doi: 10.1093/bioinformatics/btl210.

A. Hassan, J. Ahmad, H. Ashraf, and A. Ali. Modeling and analysis of the impacts of jet lag on circadian rhythm and its role in tumor growth. *PeerJ*, 6:e4877, June 2018. ISSN 2167-8359. doi: 10.7717/peerj.4877.

P. Traynard, C. Feillet, S. Soliman, F. Delaunay, and F. Fages. Model-based investigation of the circadian clock and cell cycle coupling in mouse embryonic fibroblasts: Prediction of RevErb-α up-regulation during mitosis. *Bio Systems*, 149:59–69, Nov. 2016. ISSN 1872-8324. doi: 10.1016/j.biosystems.2016.07.003.

2 Supplementary Figures

2.1 Supplementary Figure S1

```
import astrologics as ast

# Load the model path and create LogicEnsemble object
model_path = '../models/test_bonesis/'
model = ast.ensemble(model_path, project_name = 'test_bonesis')
model.create_simulation()

# Configure simulation parameters
model.simulation.update_parameters(max_time = 15, sample_count = 1000, thread_count = 15)
model.simulation.run_simulation()

# Create trajectory submodule and calculate models distances
model.create_trajectory()
model.trajectory.calculate_distancematrix(mode = 'endpoint')

# Perform MDS (Multidimensional Scaling) for visualization
model.trajectory.calculate_MDS()
model.trajectory.plot_MDS(s = 100, fig_size = (8,8))

# Calculate mode clusters
model.trajectory.calculate_kmean_cluster(n_cluster = 2)
model.trajectory.plot_MDS(s = 100, fig_size = (8,8),plot_cluster = True)

# Create logic submodule and analyze logic rules
model.create_logic()
model.logic.create_flattend_logic_clause()

# Calculate statistics between 2 model cluters and plot summary
model.logic.map_model_clusters(model.trajectory.cluster_dict)
model.logic.calculate_logic_statistic(pval_threshold = 0.0001)
model.logic.plot_logicstat_summary()

# Plot the heatmap of 'MytL1' logic rule
model.logic.plot_logic_heatmap(node = ['MytL1'], figsize = (10,8))
```

Supplementary Figure S1. An example script of AstroLogics pipeline to replicate all plots from Figure 2

2.2 Supplementary Figure S2

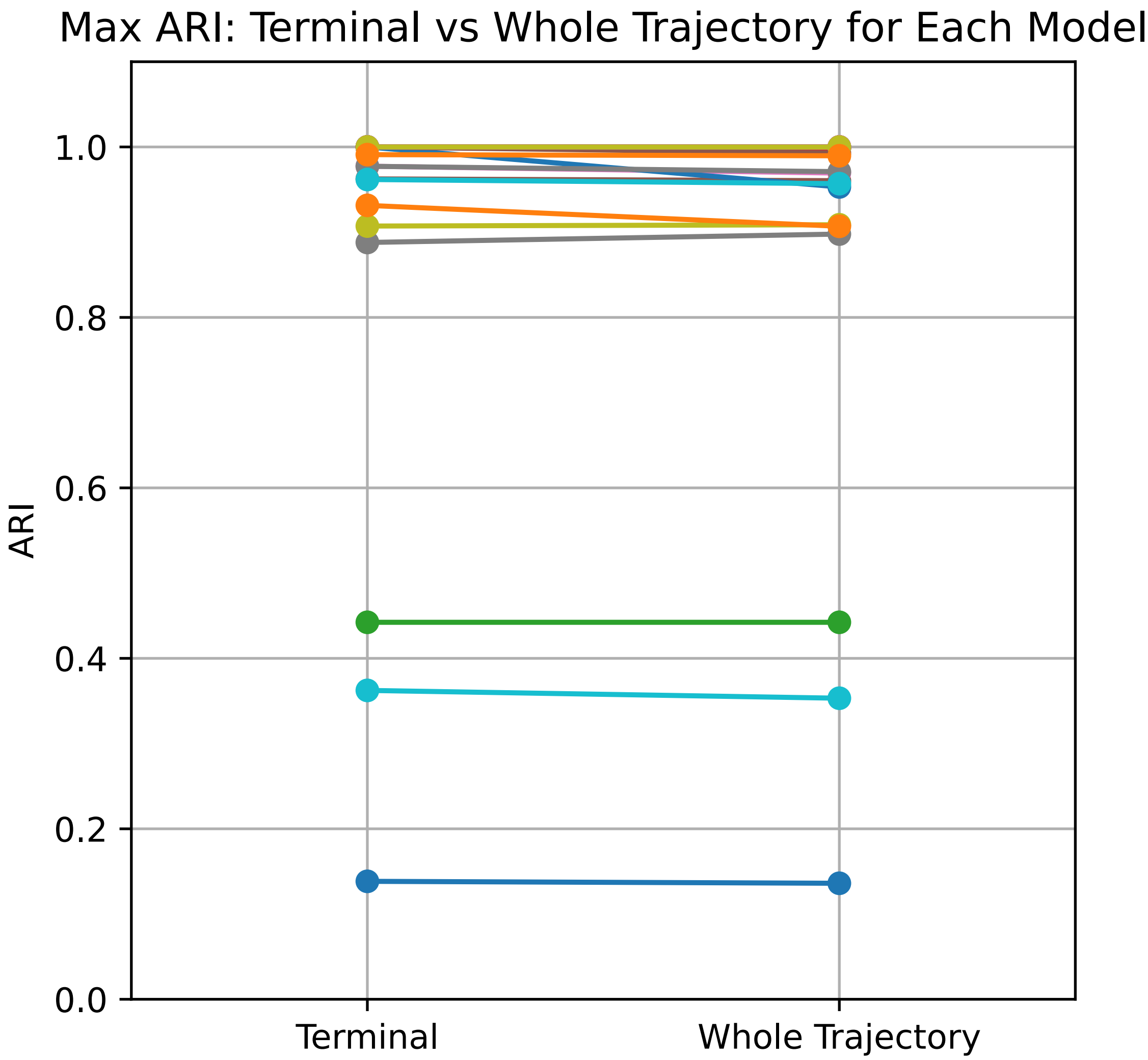

Supplementary Figure S2. A comparison of two simulation-based clustering methods, in comparison with attractor group clustering. The y-axis represents the correspondence between attractor-based and simulation-based clustering, as measured by the Adjusted Rand Index (ARI), with 1 indicating perfect agreement and 0 indicating random agreement. The x-axis displays two simulation-based clustering methods: endpoint, which utilizes the simulation data at the terminal time point, and whole trajectory, which employs the entire simulation trajectory to calculate the distance between models.

2.3 Supplementary Figure S3

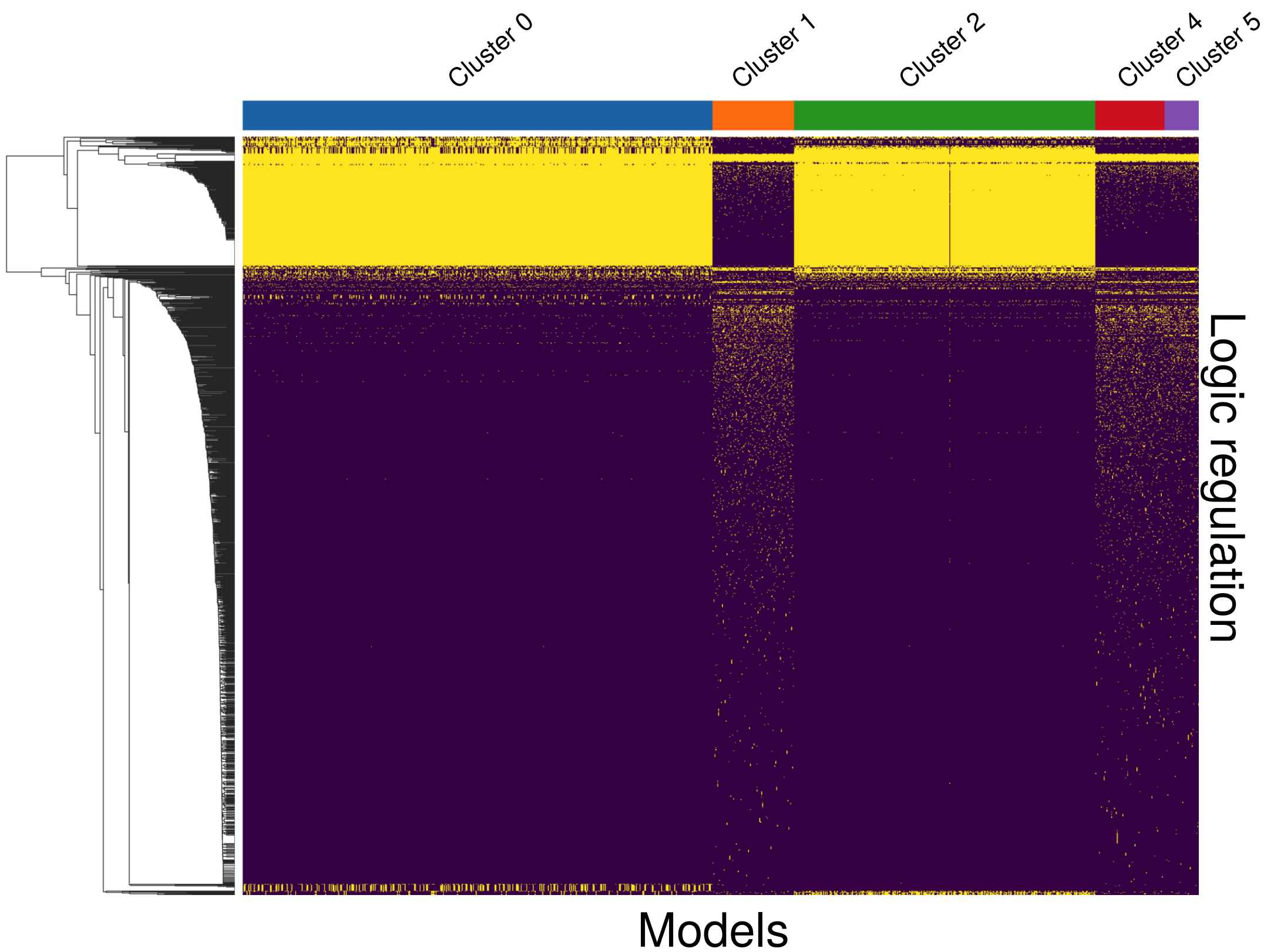

Supplementary Figure S3. Heatmap shows different logic features (row) in each BN (columns), where yellow color indicates that the BN contains certain logic features. The color bar at the top of the plot shows which clusters each BN belongs to.

2.4 Supplementary Figure S4

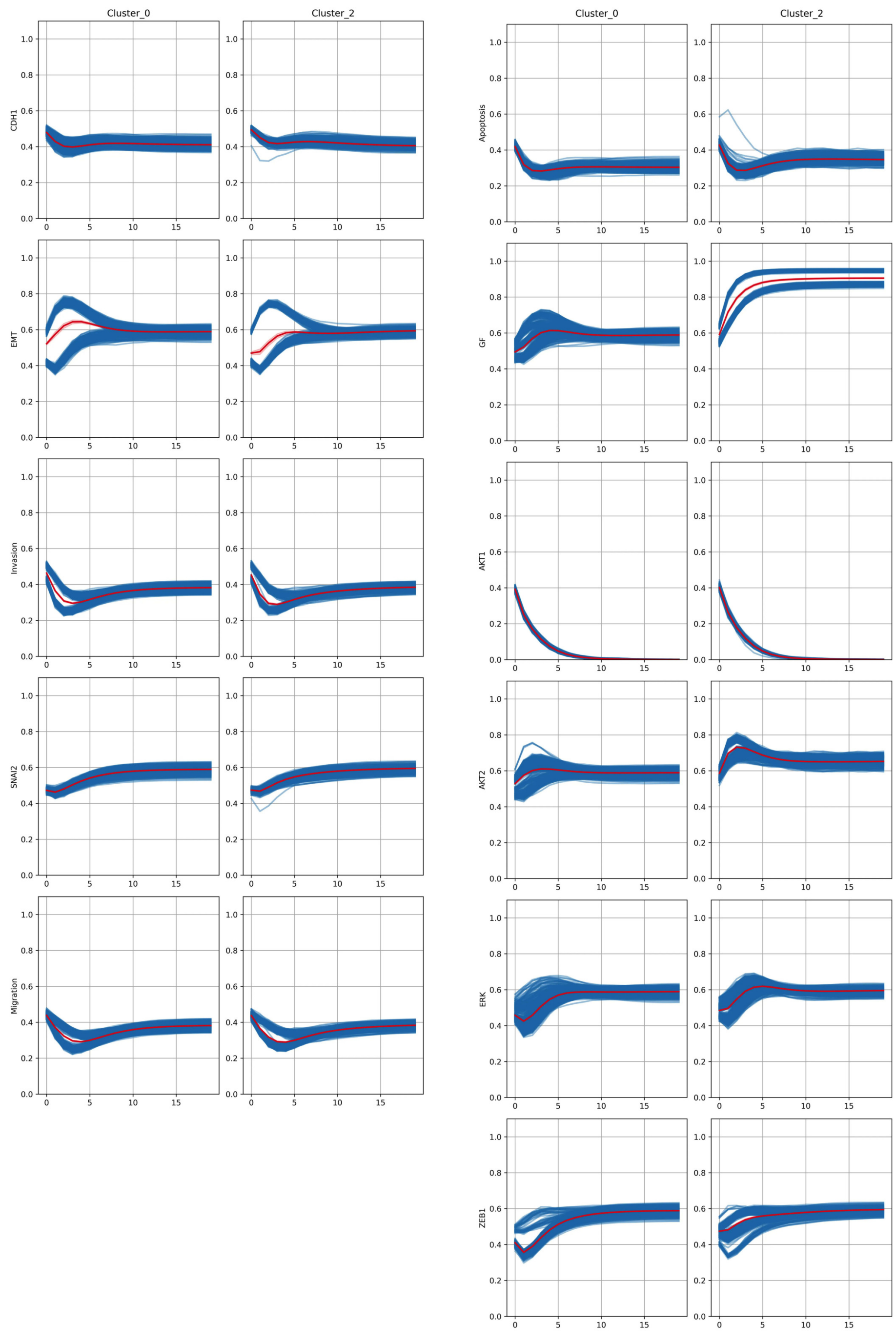

Supplementary Figure S4. Dynamics of selected node in two BN clusters from the invasion model network. Each plot shows the node activation probability of selected nodes (CDH1, EMT, Invasion, Apoptosis, GF, AKT1, AKT2, ERK, ZEB1) from the invasion model. The y-axis shows the node activation probability, and the x-axis shows the simulation time points from 0 to 20. The plot is split into two according to the BNs clusters (cluster 0 and 2).

3 Supplementary Analysis

3.1 Supplementary Analysis 1: Low ARI Scores

In this part, we performed follow-up analysis on three cases with low Adjusted Rand Index (ARI) scores when comparing attractor-based and simulation-based clustering. The analysis was conducted on two BN ensembles: Cortical Area Development (upper panel) and Human Gonadal Sex Determination (Lower panel). The HCC1954 model was excluded due to its high number of reachable attractors (approximately 18,000).

3.1.1 Analytical pipeline

- **Attractor calculation**. We first computed a set of reachable attractors for each BN in the ensemble. We then summarize them as binary heatmaps, with row colors representing the group of models that share the same set of attractors, and each column represents each attractor that the model could reach.
- **Reachability calculation through MaBoSS simulation**. We then estimate the reachability of the model by performing MaBoSS simulations from the random initial states for each BN in the ensemble. We obtained the probability of each attractor state activation probability at the end point of the simulation. We plotted this state activation probability matrix using the same row and column order as the previous matrix.
- **MDS projection of simulation distances**. We then computed inter-model distances from the terminal time point simulation data and projected them onto the MDS space. We colored each model by the shared attractor group.

3.1.2 Results

From the results, we found that the pattern of attractor activation probability follows the reachable attractors, but shows different levels of reachability, due to different STG structures. The MDS projection successfully identified major groups of models with similar reachability profiles (BN group 0 in Cortical Area Development; BN groups 0, 1, and 2, 3 in Human Gonadal Sex Determination). However, the projection failed to distinguish some clusters that differ in low attractor reachability, such as between BN groups 1, 2, 3, and 4 in Cortical Area Development, or between BN groups 0 and 1, or 2 and 3 in Human Gonadal Sex Determination.

3.1.3 Interpretation

These results explain the low ARI scores observed when comparing attractor-based and simulation-based clustering. Because AstroLogics weights models by the reachability of their attractors, subgroups that differ only in rarely reached attractors contribute little to simulation-based distances and therefore collapse in the MDS projection, even though they are distinguishable at the level of the full attractor set.

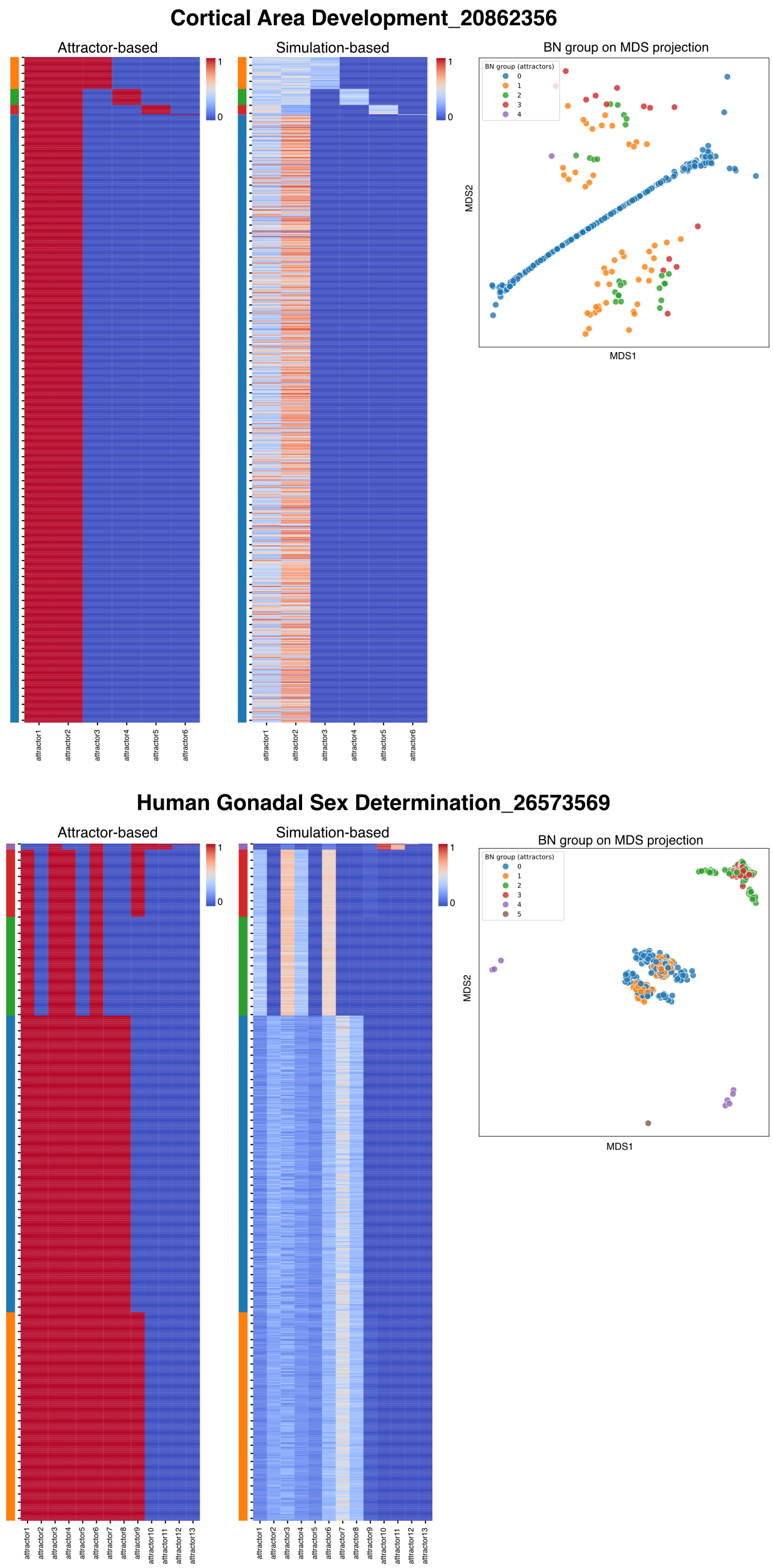

Supplementary Figure S5. The comparison of the calculated attractors (left panels), and attractor reachability (state activation probability) calculated by MaBoSS (middle panels) and MDS projection of BN distances from terminal time point simulations (right panels) of two selected model ensembles: Cortical Area Development (upper panels) and Human Gonadal Sex Development (lower panels)

3.2 Supplementary Analysis 2: Comparison with Edge-Based Methods

In AstroLogics framework, the classification of models is based on their dynamical property (e.g., sets of reachable attractors or global dynamics, as captured by approaches such as succession diagrams). In our implementation, we use MaBoSS simulations as a practical approximation of these dynamical properties.

3.2.1 Analytical pipeline

We use MaBoSS simulations to approximate each model's dynamical behavior, compute a simulation-derived distance matrix between models, and cluster models based on this dynamical distance matrix. In comparison, we process the network from each .bnet file and convert the following network into edge-based features (where activation edge is assigned a value of 1 and inhibition edge is assigned a value of -1). We then calculated the distance between models, using the Hamming-distance approach to obtain the model's pairwise-distance matrix. Finally, we compare between (i) clustering based on the simulation-derived distance matrix and (ii) clustering based on purely structural edge features, using the Death Receptor Signaling_20221256 network from Kadelka.

3.2.2 Results

In the left panel, we report the clustermap of MaBoSS-based model distances, with row/column annotations indicating the reachable-attractor sets for each model. In the right panel, we report the corresponding clustermap obtained from a Hamming-distance matrix computed on edge features, using the same attractor-set annotations.

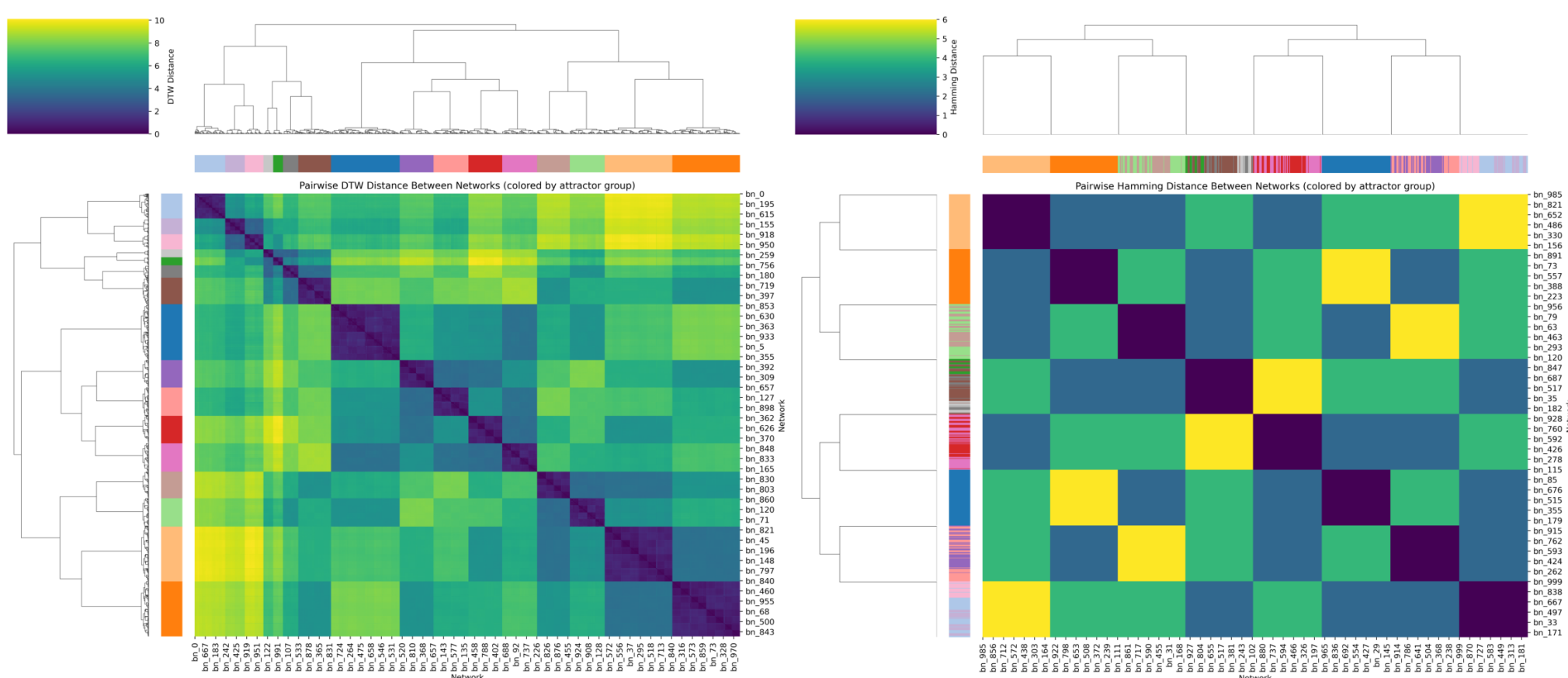

Supplementary Figure S6. Comparison of distance matrix between MaBoSS-derived (left panel) distances and edge-based distances (right panel).

3.2.3 Interpretation

This comparison shows that MaBoSS-derived distances more faithfully separate models according to their reachable-attractor sets than edge-based distances. The script to this analysis is available on GitHub: `/scripts/Results_Model_classification_comparison`.

3.3 Supplementary Analysis 3: Convergence Analysis

Reporting a convergence assessment is important, since the node activation probabilities (and the downstream inter-model distances used for clustering) are estimated by Monte Carlo sampling. In the AstroLogics framework, node activation probabilities are estimated by Monte Carlo sampling through MaBoSS. Due to the importance of node activation probabilities in defining downstream inter-model distance and clustering, we decided to include a lightweight convergence check designed to quantify sampling error. The function is implemented in the `model_simulation.py` under the function name `.simulation.analyze_sample_count_convergence()`. We demonstrate the use case of this function in the Invasion model tutorial of this work.

3.3.1 Analytical pipeline

In this function, we randomly select a subset of models. We repeated MaBoSS simulations with increasing numbers of sample counts (e.g., 100, 250,...,5000) and monitored the stabilization of the simulation endpoint, node activation probability through the mean standard deviation (mean std). We define a threshold parameter, `tolerance`, with the default value of 0.01. The suggested number of sample count is where the level of mean std is lower than the `tolerance`. In this example, we sampled 15 models from the model ensemble and performed 5 simulation replicates.

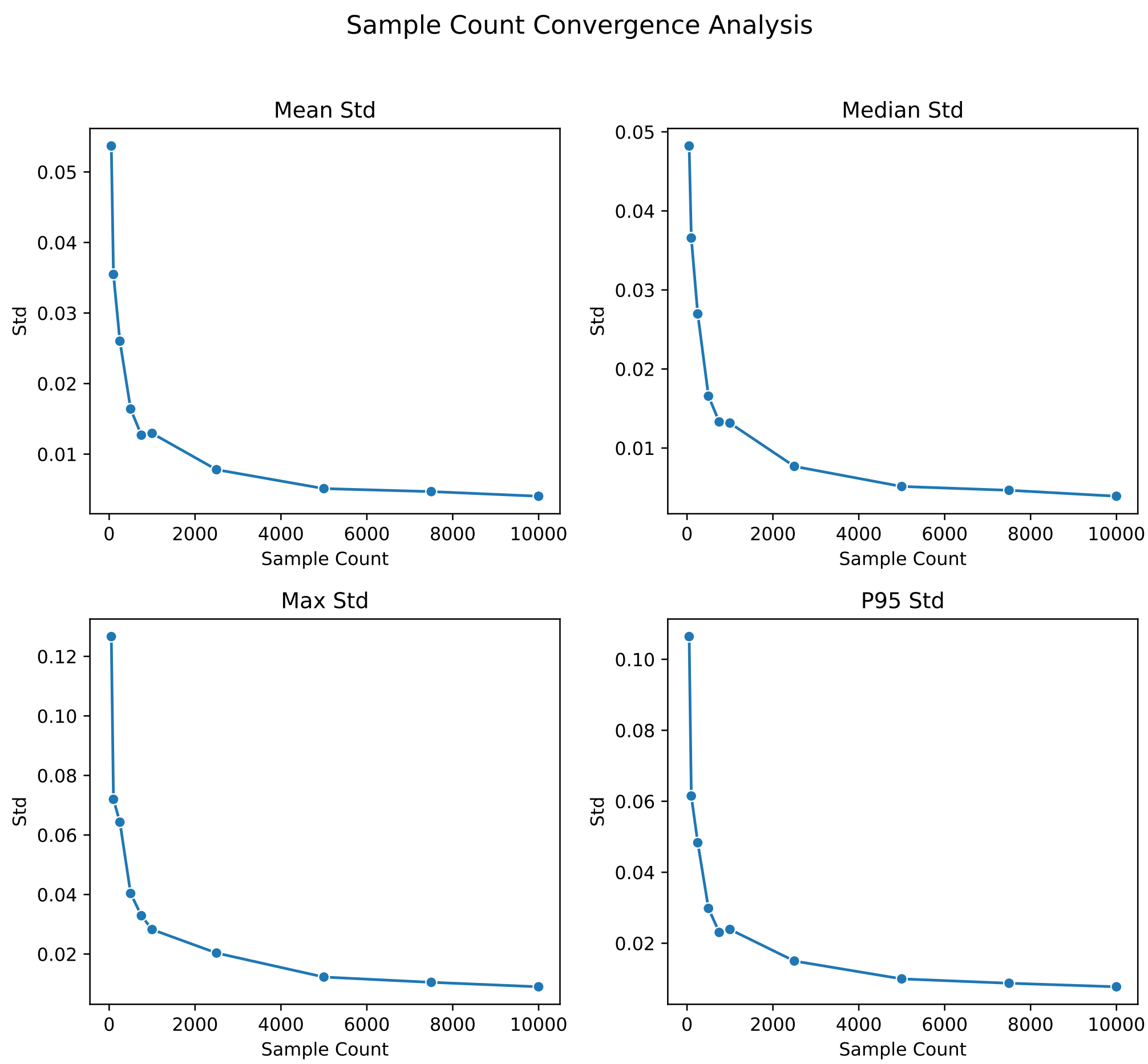

Supplementary Figure S7. Convergence analysis of node activation probabilities for the Invasion model. Each plot shows different metrics of the standard deviation on the y-axis(Mean, Median, Max, and p95, accordingly, from left to right, top to bottom), while the x-axis shows the number of samples.

3.3.2 Results

The convergence analysis reveals that the node activation probabilities stabilize after around 1000-2000 sample counts, indicating sufficient sampling for reliable estimates. The suggested sample count is determined based on the stabilization of the mean standard deviation, ensuring robustness in the downstream clustering analysis. We therefore used the sample count of 2000 for our downstream analysis of in the Invasion model tutorial.
